# Grade nine learners’ experiences on Ubuntu workshop in Mopani District of South Africa: An appreciative inquiry

**DOI:** 10.4102/hsag.v30i0.2884

**Published:** 2025-05-31

**Authors:** Pride Bvukeya, Katekani J. Shirindza, Rachel T. Lebese

**Affiliations:** 1 Department of Advanced Nursing Science, Faculty of Health Sciences, University of Venda, Thohoyandou, South Africa; 2 Department of Public Health, Faculty of Health Science, University of Venda, Thohoyandou, South Africa

**Keywords:** appreciative inquiry, experiences, grade nine learners, programme, teenage pregnancy, Ubuntu

## Abstract

**Background:**

Teenage pregnancy remains a significant public health concern in South Africa that remains a challenge in the context of Ubuntu in the African philosophy.

**Aim:**

This article explores grade nine leraners’ experiences on Ubuntu workshop in Mopani District.

**Setting:**

The study was conducted in Limpopo province of South Africa. Limpopo province is one of the nine provinces on the far northern side of South Africa. It is bordered by Mozambique and Zimbabwe as its neighbouring countries.

**Methods:**

A qualitative exploratory descriptive design was used based on the 4-cycle appreciative inquiry. A non-probability purposive sampling was used to select 32 participants who were grade nine learners who participated in the health education programme for teenage pregnancy. Data were gathered through workshop group discussions. The interviews were audiotaped and transcribed verbatim. Thematic analysis was used to categorise and analyse themes that emerged from the data.

**Results:**

Four main themes were identified: Empowering self and others; Outcomes for the future; Imparting knowledge; and Shaping others.

**Conclusion:**

Based on the four themes that emerged from the findings, it is clear that participants prioritise personal growth, empowerment and positively impacting others. These findings suggest that teenage pregnancy prevention programmes should focus on empowering teenagers to empower others and that knowledge sharing with the infusion of Ubuntu principles promotes self-worth, dignity and empowerment, enabling teenagers to make informed decisions about their reproductive health.

**Contribution:**

Adds to the body of knowledge by presenting Ubuntu teenage pregnancy prevention programmes.

## Introduction

South Africa, like many other countries, faces significant challenges in addressing teenage pregnancy. According to the *South African Schools Act* 84 of 1996., approximately 100 000 teenage girls drop out of school each year because of pregnancy (Mbongwa, Mpanza & Mlambo 2024). This affects the educational and economic prospects that perpetuate cycles of poverty and inequality (Le Roux 2020). Significant strides have been made in addressing teenage pregnancy in South Africa, such as the Parent Centre’s Teen Parenting Programme to provide teenage parents and caregivers with support, parenting skills and life skills to help them cope with their responsibilities. The National Teenage Pregnancy Partnership (NTPP) is a coalition of Non-governmental organisation (NGOs), government departments and university partners working together to reduce teenage unintended pregnancy in South Africa. Teenage pregnancy prevention programme is an initiative that aims to reduce the number of pregnancies amongst adolescents, typical ages 13 years old–19 years old (Kassa et al. 2018). These programmes often provide a comprehensive approach to prevention, including education about sexual health and access to contraception. It also empowers teenagers to make informed decisions through effective communication, thus developing a healthy relationship. Historically, the teenage pregnancy prevention programme entailed a range of strategies and activities to reduce teenage pregnancy (Kassa et al. 2018).

Over the change of time, the programmes have evolved to address the changing societal needs and the best practices, as evidenced through research projects (Kassa et al. 2018). In various South African high schools, various teenage pregnancy prevention programmes were found to vary depending on the district and the province of South Africa. According to Barron et al. (2022), numerous intervention programmes have been established throughout KwaZulu-Natal; nevertheless, the birth rate rose in every state. In Zambia, the sex education programme did not encourage teenagers to avoid early sexual behaviour; hence, the pregnancy rate increased by 29% to 48% (Malunga et al. 2023). In South Africa, the intervention strategy for teenage pregnancy displayed low uptake of contraceptives, thus increasing the number of teenage pregnancies (Ntini et al. 2023).

The study conducted by Mosalagae (2023) reflected that Ubuntu philosophy within geospatial Africa is a dominant ontological system of humanness used to bring all cultures together through its principles. The concept of Ubuntu as an African philosophy underpins holistic care within communities (Ajitoni 2024). According to Adewale (2023), it was revealed that it is imperative to integrate the Ubuntu philosophy within the teenage pregnancy prevention programme in high schools. The infusion of Ubuntu’s philosophical values and principles has, for a long time, played an integral part in curbing poor health practices whilst promoting health (Adewale 2023). However, there are still challenges, such as attitudes, age disparity, psychological status, peer pressure, socio-economic status, the exploratory attitude of learners, media, lack of role models, previous experiences, socio-economic status and lack of parental love, within the high schools of Mopani District that hinder the implementation of the teenage pregnancy prevention programmes within the communities (Adekola & Mavhandu-Mudzusi 2021). In support of Mosalagae (2023), Adewale (2023) and Ajitoni (2024), the infusion of Ubuntu principles and values can be integrated into the teenage pregnancy intervention programme to reduce the rate of teenage pregnancy that increased by 17.9% in selected high schools of Mopani District, Limpopo province (Risenga & Mboweni 2022).

According to the study conducted by Miyanda and Wakunguma (2014), an Ubuntu-centred consensus in a teenage pregnancy prevention programme should prioritise collective responsibility in involving the community, parents and all teenagers in shared decision-making. There should be interconnectedness between parents and community stakeholders that would have an impact on the teenagers’ actions within the community. Several studies were conducted to reflect the relevancy of the programmes. Lohan et al. (2018) indicated that in the United Kingdom, there has been an effective programme that was relevant and displayed positive results in the reduction of teenage pregnancy, especially when males are involved. Shirao, Momanyi and Anyona (2020) also indicated that some programmes were available only to increase the knowledge and skills of teenagers, but according to Brindis et al. (2020), such programmes have little direct impact on the values, attitudes and sexual behaviour of teenagers. This shows that the programmes need the involvement of all genders with the infusion of Ubuntu for effectiveness towards its implementation.

Therefore, it is crucial to implement a teenage pregnancy prevention programme with the infusion of Ubuntu principles and values to convey educational health messages to the teenagers within the selected high schools.

### Research aim

The study aims to explore the experiences of grade nine learners on the attendance of Ubuntu-infused pregnancy prevention workshops in Mopani District, South Africa, using an appreciative inquiry (AI).

## Research methods and design

### Study design

A qualitative, exploratory and descriptive design was used to explore and describe the experiences of grade nine learners on the attendance of Ubuntu-infused pregnancy prevention workshops in the selected high schools of Mopani District, Limpopo, using an AI. Appreciative inquiry is more concerned with building on the strengths and opportunities of the phenomenon studied, rather than focusing on the problems (Trajkovski et al. 2013). It emphasises offering a flexible opportunity to facilitate change from the grassroots upwards (Spofford 2017).

#### Study setting

The study was conducted in the selected high school of Mopani District in Limpopo province of South Africa. The high school is located in the Mopani East district at Hlaneki village, Mopani District of Limpopo province. Hlaneki village falls under the Greater Giyani Local Municipality, which is located in the Mopani District Municipality, Limpopo province of South Africa. The municipality is known for its rich cultural heritage and diverse population, with Xitsonga being the most spoken language in the area. It is situated in a rural setting that serves the local community, providing secondary education to learners from Hlaneki village and the surrounding areas. The school had a total of 952 learners and a student ratio of 31:1. The teenage pregnancy amongst adolescent girls aged 15–19 years increased by 17.9% in the rural area of Hlaneki village.

**FIGURE 1:**
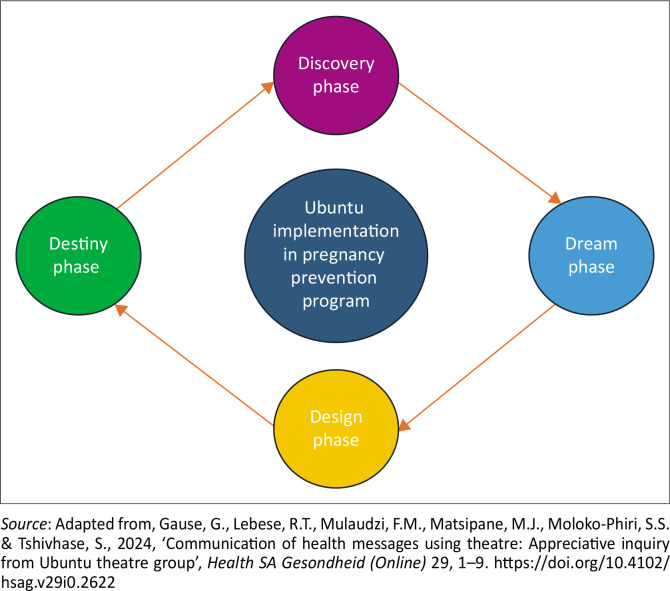
4-D cycle of appreciative enquiry.

As a result, Ubuntu booklets (University of Venda 2024) with designed scenarios were used to convey valuable information to the teenagers during the workshop training. The training programme includes Ubuntu, a Nguni Bantu term meaning humility. The values of Ubuntu include compassion, humility, solidarity, equity, mutual responsibility, social justice, communalism and interdependence. Ubuntu promotes a spirit of community over individualism. The programme is implemented for the grade nine learners of the selected high school of Mopani District, educating them about teenage pregnancy prevention with the infusion of Ubuntu Nursing Model. The Ubuntu Nursing Model was funded by the National Research Foundation by Prof Mulaudzi from the University of Pretoria in collaboration with the University of Venda and North-West University. The workshops were conducted four times in a year, with stakeholders from the chieftaincy, parents and grade nine learners from the selected high school of Mopani District. The training programme was funded by the Community Engagement office of the University of Venda.

#### Population

The population in this study comprised grade nine learners from a selected high school in a rural province of South Africa. The group comprises 32 grade nine learners who were involved in the workshops regarding the prevention of teenage pregnancy within the Ubuntu perspective.

#### Sampling

A non-probability purposive sampling method was used. The sampling method was used to provide rich data from the participants about the implementation of teenage pregnancy prevention programmes through consensus. All learners from grade 9 who fit the inclusion criteria were enrolled in the programme. Two workshops were conducted for the participants, and data collection was conducted after the participants attended both workshops. A sample of 32 participants from grade nine learners of the selected high school of Mopani District was used as enrolled in the programme. The inclusion criteria were a teenager, both males and females aged between 15 years and 19 years who were in grade nine at the selected high school of Mopani District. The size of the sample depended on data saturation. The sample of participants was divided into four focus groups, with an average of eight participants per group.

### Recruitment of participants

Grade nine learners were recruited from the selected high school of Mopani District, Limpopo province. The meeting was arranged with the school principal and teachers from the selected high school of the Mopani District. The principal linked the researcher with the school teachers within the school environment, who arranged the initial recruitment of grade nine learners by linking them to the researcher. Appointments were made with parents of grade nine learners to share information about the study and to secure informed consent and assent from the grade nine learners, as most of them were underage. After a thorough explanation with the parents of the participants regarding the study, the purpose of the study, the procedures involved, the potential benefits and risks, how confidentiality will be maintained and their right to withdraw from participation, the informed consent form was ensured. Participants and parents of the grade nine learners made an informed decision regarding participation in the study. The researchers’ explanation was at the level of the participants’ understanding.

### Data collection

Data collection was carried out at the selected high school of Mopani District where participants were recruited for the study. Two workshops were conducted, one before data collection and the other followed by an exploration of grade nine learners regarding their experiences on the implementation of the teenage pregnancy prevention programme. Data were collected by two researchers using focus group discussions through two workshops that were conducted in the form of scenarios. Focus group interviews were conducted after school and on Saturdays, as it was convenient for teenagers attending school. The interviews were conducted in Xitsonga with grade nine learners at the identified church building within the school premises with enough space. The focus group interview guide was written in English and translated into Xitsonga by the language experts to allow the free expression of participants during the focus group interview. The questions that were used to guide the focus group discussions were described by the 4-D of AI. The participants were asked to describe their best experience of the Ubuntu-infused teenage pregnancy prevention programme, to share the preventive teenage pregnancy information from an Ubuntu perspective, to describe their ideal image of using the programme to reach consensus to avoid teenage pregnancy, to describe the processes that should be in place to support the reduction of pregnancy rate by participants and to describe the measures that should be used to sustain the effectiveness of the implementation of the teenage preventive programme from an Ubuntu perspective. The focus group interview was conducted in a conducive environment free from noise. Written consent forms were handed out to be signed before commencing the focus group interview. A flip chart board and a pen were provided by the researchers. Participants were allowed to stand or adopt any position when they wished to emphasise something. During the focus group interviews, the researchers took field notes. The research assistant operated the audiotape, which was placed in the middle of the circle to capture every participant’s voice. The voice recorder was used with the permission of the participants.

### Data analysis

Deductive thematic content analysis was used to enable the researchers to rigorously analyse data and draw meaningful conclusions that contribute to practical applications. Data were analysed independently by the two researchers in this study, who met and discussed the findings and agreed with the developed themes and sub-themes. Data analysis was conducted according to the process of Tesch’s eight-step method (Creswell & Plano Clark 2023). The method of open coding was described according to the following points:

- Get the sense of the whole: The researcher read all transcriptions carefully and jotted down ideas that came to the researcher’s mind.
- Pick one document: The researcher picked one transcript and went through it to see what it is all about. The researcher highlighted the meaning and wrote her thoughts down the margin.
- List the topics: The researcher clustered similar topics together and arranged these groups in columns under major, unique topics and leftovers.
- Go back to data: The researcher abbreviated the topics as coded and wrote the codes next to the appropriate segments of text to see whether new categories and codes emerge.
- Describe the topics: The researcher found the most descriptive word for each topic and turned them into categories, and related topics were grouped together to reduce the number of categories.
- Abbreviate categories: The researcher made a final decision about the abbreviations for each category and alphabetised the codes.
- Assemble the data: The researcher gathered data that belong to each category and did a preliminary analysis.
- Recording: The researcher recorded the data and reached consensus with the independent co-coder on themes.

### Measures to ensure trustworthiness

Trustworthiness is a method of establishing rigour in qualitative research without sacrificing relevance. Trustworthiness was ensured throughout the study. The four criteria that were used to ensure trustworthiness were credibility, transferability, dependability and confirmability (Lincoln & Guba 1985). To ensure credibility, data triangulation was ensured by using different data-collecting methods. The researchers collected data through observational notes and focus group discussions with participants. Transferability refers to the degree to which the findings can be applied to another context and setting or with other groups (Lincoln & Guba 1985). Transferability was ensured through a detailed description of the research context and setting to allow others to assess how transferable the findings were. The purposive sampling technique was used to select participants who fitted the criteria described. The results with supporting direct quotes from participants were also described densely. Dependability was established through the use of experienced researchers who analysed and coded data, and, lastly, confirmability was ensured through the dense description of the research methods employed throughout the manuscript.

### Ethical consideration

An application for full ethical approval was made to University of Venda and University of Venda Research and ethics consent was received on 19 August 2024 (ethics clearance no.: FHS/21/PH/17/1310). Parents signed the consent form, and learners aged 15 years – 17 years signed the assent form to participate in the study. Participants were assured of their anonymity and confidentiality, as all participants used code names which were difficult to identify and match to a specific participant. Participants were further assured of their voluntary participation, as they were allowed to withdraw at any point of the study without any penalty. The independent coder and all researchers signed confidentiality agreements.

### Presentation of findings

Focus group interviews were conducted with 32 participants attending school at the selected high school of Mopani District. The focus group discussions were conducted at the church building next to the selected high school. The results of the study are guided by the 4-Ds of AI design, including the discovery, dream, design and destiny phases with the inclusion of the demographic characteristics of the participants. Table 1 shows the demographic data of the grade nine learners who participated in the study.

**TABLE 1:** Demographic characteristics of participants (*N* = 32).

Focus group no.	Total number of participants	Age range of participants	Gender		Language used
			Male	Female	
1	8	15–19	4	4	Xitsonga
2	8	15–18	4	4	Xitsonga
3	8	15–17	2	6	Xitsonga
4	8	15–19	-	8	Xitsonga

### Narrative description of the demographic characteristics of the participants

Table 1 reflects the participants involved in the study and the number of people who participated in the study by means of focus group discussions. The participants who were included in the study were both male and female grade nine learners. Thirty-two participants were involved in the focus group discussions conducted. The ages of participants ranged from 15 years to 19 years.

Amongst the participants, 68.7% (22) of participants were females and 31.2% (10) were males. The highest number of participants were those at the age of 15, comprising 31.2% (10) of learners; 16-year olds were 6.2% (2), those aged 17 years were 37.5% (12); 18 years were 9.37% (3); and 19 years were only 15.6% (5) of learners. All ethnic groups were included within the group of participants, with 68.7% (22) of Xitsonga-speaking participants, 12.5% (4) of Sepedi-speaking participants, 15.6% (5) of Venda-speaking participants and 3.1% (1) of Xhosa-speaking participants, but all were fluent in the Xitsonga language. All participants agreed to participate in the Ubuntu research project at school using the Xitsonga language.

Four main themes emerged from the discussions according to the phases with the sub-themes. The main themes are empowering self and others, outcomes for future roles, imparting of knowledge and shaping others, which were identified in this study, and each had its sub-themes. The themes and sub-themes are displayed in Table 2.

**TABLE 2:** Themes and sub-themes.

Themes	Sub-themes
1. Discovery phase: Empowering self and others	1.1 Training workshops 1.2 Considering risk factors and vulnerabilities 1.3 Setting boundaries and informed decisions
2. Dream phase: Outcomes for the future	2.1 Education and awareness 2.2 Skills building 2.3 Support networks 2.4 Healthy relationships
3. Design phase: Imparting knowledge	3.1 Implementation of peer education 3.2 Development of youth group
4. The destiny phase: Shaping others	4.1 Leadership skills 4.2 Community engagement 4.3 Advocacy skills

### Presentation of findings

The findings are discussed according to the participants, as reflected from the themes and sub-themes based on the AI. It was also noted with concern that teenagers mentioned the benefits of the teenage pregnancy prevention programme related to the four phases of the AI. The phases are the discovery phase, the dream phase, the design phase and the destiny phase. From each phase, one theme emerged with sub-themes identified from the theme. The following are the phases with themes and sub-themes that were reflected from participants during focus group discussions:

- Discovery phase: Empowering self and others,
- Dream phase: Outcomes for the future,
- Design phase: Imparting knowledge,
- Destiny phase: Shaping others.

### Theme 1: Discovery phase: Empowering self and others

The discovery phase explored the participants’ expressions of the consensus to reach after receiving the training programmes. The main theme that emerged from the discovery phase is the sense of empowering self and others. Participants expressed that there is a need to develop a sense of empowering themselves and others within the community and to set boundaries amongst themselves as teenagers. The boundaries will assist them in making informed decisions on matters related to sexual relations. The participants also discovered that there is a need to communicate openly with peers, family and healthcare providers regarding the implementation of information received from the training workshop. The following are the sub-themes that emerged, as displayed in Table 2.

#### Sub-theme 1.1: Training workshops

Participants felt that the workshops that were conducted were beneficial to their well-being, and hence they felt strengthened with the information that was presented:

‘I think the workshop is meant to empower us as teenagers to teach others at schools so that they can have a better future in our lives.’ (Participant 7, female, 18 years in focus group 1)

Participants displayed increased confidence through training workshops:

‘I learned so much about my reproductive health and I am now comfortable to talk about sex and relationships with my friends and my family members.’ (Participant 4, female, 16 years in focus group 1)

‘I realised that I have an improved self-awareness, hence I gained a better understanding of strength, weakness and values in self.’ (Participant 8, male, 15 years in focus group 1)

This study shares the effectiveness of teenage pregnancy prevention programmes, which predominantly educate teenagers in the selected high school of Mopani District, Limpopo province. Whilst the research is confined to one selected high school, the results indicate the importance of teenage pregnancy prevention programmes within high schools. In consistent with the results of the previous study that assessed the impact of teenage pregnancy prevention programmes, Pretorius and Plaatjies (2023) identified that self-awareness was considered a key emotional intelligent skill for secondary school principals’ leadership toolkit. Through training workshops provided to school principals, they considered that self-awareness increased emotional awareness, self-regard, self-confidence, assertiveness and independence. This shows that workshops and seminars were strongly recommended, as they contributed to increasing the success rate amongst school principals. Our analysis highlighted that a goal of self-awareness is that of developing self-knowledge and understanding to assist in personal development (Ashley & Reiter-Palmon 2012). This is an indication of how grade nine learners understood the importance of training workshops that were conducted, thus providing them with the success of their self-awareness regarding their aspects of human development.

#### Sub-theme 1.2: Considering risk factors and vulnerabilities

Participants indicated that there is limited knowledge about reproductive health, which is aligned with inadequate parental guidance and peer pressure; hence, they regard it as a risk factor and vulnerability to teenagers’ sexuality and reproductive health. The following statements reflected quotes from participants:

‘I never knew that not having a support system could increase my risk of getting pregnant as a teen. Hence, I am confident that with the knowledge gained in this workshop, I am very sure that I am surrounded by people with positive minds.’ (Participant 4, female, 16 years in focus group 1)

‘I also realised that I was a vulnerable group to peer pressure of which by then I didn’t know about it. With this workshop, I truly accept that my eyes are widely opened and will therefore make a valid decision with my life and those of others in my family.’ (Participant 2, female, 15 years in focus group 1)

The overall impact of the teenage pregnancy prevention programme experienced by teenagers in the current study is almost similar to the study investigating teenage pregnancy training workshops in Indonesia (Effendi et al. 2021). According to the previous study, it was discovered that the teenage pregnancy prevention training workshop was the best intervention for adolescents, as they discovered factors that were detrimental to their lives (Effendi et al. 2021). However, it was found that in resource-constrained communities or nations like Ghana, lack of teenage pregnancy prevention programmes jeopardises adolescents education and employment opportunities, leading to their life’s vulnerabilities. As a result, girls below the age of 18 years who become pregnant were faced with higher chances of facing violence in a marriage or partnership, leading them to the risks of vulnerability in their real lives (WHO 2020). The study conducted by Ramírez-Villalobos et al. (2021) alluded to the inclusion of training programmes in sexuality education improving teachers’ knowledge about sexual and reproductive health. The students who received counselling from teachers about sexuality and reproductive health were fully protected from vulnerabilities; hence, they were able to know the risk factors associated with teenage pregnancy. This was supported by Philibert and Lapierre (2023) in the study conducted regarding strategies for preventing teenage pregnancy. The study concluded that there are positive results when teenagers are provided with information during the teenage pregnancy prevention programmes, thus protecting them against the vulnerabilities and risk factors of teenage pregnancy.

#### Sub-theme 1.3: Setting boundaries and informed decisions

Setting boundaries and making decisions refer to the ability to identify and communicate limits and needs as well as prioritising well-being and safety (Levin 2021). Participants described how they would make their own choices with their own values and goals that may boost their body image:

‘I realised that my choices were not reflecting my own values when it comes to teenage pregnancy. I was so lost without any guidance from people who were not giving me information about sex. Through these workshops, I feel confident and empowered that I can also lead others in the right direction.’ (Participant 6, female, 16 years old in focus group 2)

Teenagers cited that they were very lost when it came to making decisions regarding their sexuality. They reflected that their choices were negatively affected, as there was nobody to guide or support them. Teenagers felt that the issue of setting boundaries and making informed decisions gained from the workshop made them prioritise the sexuality issues. The overall impact of the teenage pregnancy prevention programme experienced by teenagers in the current study is almost similar to the study conducted in Ethiopia by Mezmur, Assefa and Alemayehu (2021). Over and above, the teenage pregnancy prevention programme has positioned teenagers to make their own choices and be able to prioritise their plans in pregnancy planning. From what has been said, it was apparent that teenagers relied on setting boundaries for most of the strategies to avoid teenage pregnancy. It should, therefore, be noted that if choices were made regarding teenage pregnancies, there would be the best deal of preventing teenage pregnancy, thus avoiding sexual health risks.

### Theme 2: Dream phase: Outcomes for the future

This is the phase in which participants imagine the ideal solutions or outcomes that might assist them in the future. Participants felt that there is a need to empower themselves through leadership roles within the group to mentor others so that they can have direction and purpose for their future careers. The following were sub-themes that emerged from the theme:

#### Sub-theme 2.1: Education and awareness

Participants reflected that in the dream phase, they promised to develop accurate information about reproductive health through education that would make them aware of contraceptives and the establishment of healthy relationships towards the teenagers of the opposite sex. The following are the quotes from the participants:

‘Now that I have learned that knowledge is power, I will empower other teenagers with information related to sexuality so that their future can be unlocked.’ (Participant 7, female, 16 years in focus group 3)

‘During the training workshop, I was coached to such an extent that I will be able to set my goals for the future and will stay accountable to all my actions of sexuality in life. I will learn to agree and disagree when I am forced to have sexual relations by force.’ (Participant 5, male, 19 years in focus group 1)

From what has been said by participants, it was obvious that teenagers gained too much information about the teenage pregnancy prevention programme. It should therefore be noted that if education and awareness regarding teenage pregnancy prevention were done, it would be a good match for teenagers to avoid teenage pregnancy and be able to seek appropriate life choices. The study conducted by Worku et al. (2021) attested that in East Africa, educating people and making them aware of factors associated with teenage pregnancy made teenagers aware of contributory factors to teenage pregnancy and the education about it. The curriculum in the South African schools does not put pressure on the schools to teach sexual health, as there are no examinations, and sexual health is not compulsory (Msutwana 2021). This shows that the teenage pregnancy prevention programme has a positive impact on sexual health education to decrease the high rate of pregnancies at schools, but according to Adekola and Mavhandu-Mudzusi (2022), the positive influence of sexuality education on young people was mostly realised in urban and semi-urban areas, whilst its impact is limited amongst high schools in rural areas.

#### Sub-theme 2.2: Skills building

Participants described skills building as an essential aspect of personal growth and development. They described how the training helped them with communication skills, leadership skills and conflict resolution skills that would assist them in articulating thoughts and needs as well as the boundaries during the training workshop. The following are the quotes from the participants:

‘I know that communication is good because I now feel more confident in expressing myself during training workshop.’ (Participant 6, female, 17 years old in focus group 4)

‘Leadership skills have empowered me to take charge of my life and this will enable me to inspire others through further training.’ (Participant 3, female, 18 years old in focus group 4)

Research shows that teenagers are much more likely to develop romantic relationships with people of the opposite sex, thus helping them to establish good communication skills during the establishment of a romantic relationship (Hielscher et al. 2021). This can be said to be a contributory aspect to the development of healthy relationships with people of the opposite sex during the teenage pregnancy prevention programme.

#### Sub-theme 2.3: Support networks

Support network refers to the people and available resources available to participants to help them grow, learn and succeed in a training workshop. This means that for an effective teenage pregnancy prevention programme to be effective, teenagers should get support from available members of the community to strengthen them. The networking with stakeholders within the community or family members would bring a huge change to teenagers; hence, it would make them bring a positive change to their lifestyles. This was reflected by one of the participants who said:

‘The support network we have built in this program has been a game-changer to me. This program has shown me that I’m not alone in my struggles and the support network provided me with a sense of belonging and connectivity with others.’ (Participant 3, male, 15 years in focus group 3)

The presence of family members or community stakeholders could result in positive results for teenagers within the programme. This means that the teenagers would, therefore, have access to resources for the prevention of further pregnancies, and hence they will be supported by the family support system or the community members. This study explored the support received from support networks by teenagers during the teenage pregnancy prevention programme. These findings concurred with the findings by Luttges et al. (2021), who reflected that teenagers who happened to be pregnant for the second time needed support structures to keep track of their careers in life. This showed that support structures in the form of reproductive clinics and preschools and the development of policies at schools would contribute to supporting teenagers who could be pregnant for the second time to continue with schooling and also take care of their children. Teenagers were therefore urged to invite parents so that they could take up their role as support networks and inform their teenagers about teenage pregnancy prevention whilst still at school.

#### Sub-theme 2.4: Healthy relationships

The participants described the benefits of the teenage pregnancy prevention workshop and reflected the impact on promoting moral development and responsible behaviour among young people in their improved knowledge and skills about sexuality and reproductive health issues. Participants demonstrated that they developed a sense of mutual respect and trust in their relationships with people of the opposite sex.

This was reflected by a participant, who said:

‘I learned to communicate effectively with my partner and will therefore set boundaries for the two of us as well as with teaching others to prevent unintended pregnancy.’ (Participant 5, female, 16 years in a focus group 3)

Another participant said:

‘I learned that healthy relationships are built upon the issue of equal power and open communication between people. Therefore, I promised that I will not allow myself or force my partner to engage in sexual relations without reaching consensus.’ (Participant 6, female, 18 years in focus group 4)

Teenage pregnancy prevention programmes play a crucial role in promoting healthy relationships amongst teenagers by equipping them with essential knowledge about responsible decision-making, communication and emotional well-being. These programmes provide young individuals with the tools to understand, consent, set personal boundaries and engage in respectful partnerships. By addressing the social, emotional and health aspects of relationships, such initiatives help teens develop mutual respect, trust and a sense of responsibility, ultimately reducing the risks associated with early pregnancies and ensuring a brighter future for young people. The findings were supported by Hielscher et al. (2021), as the training programmes have positioned teenagers in healthy romantic relationships. This shows that teenagers would agree to allow pregnancy to take place only through agreement between themselves.

### Theme 3: Design phase: Imparting knowledge

This is the phase in which participants bring in a plan to consider to implement what was planned. The participants in this phase planned to design plans that would assist with imparting knowledge to others. The following are the sub-themes that emerged from the data:

#### Sub-theme 3.1: Implementation of peer education

Participants indicated the need to share the knowledge gained with friends and community members during the training programme to ensure that there should be a reduction in teenage pregnancy within the community. The participants shared the following quotes:

‘I will try by all means to teach my peers because the best teachers are those who have been in our shoes.’ (Participant 8, female, 15 years in focus group 4)

‘I think the best transferring of knowledge is when peers are giving the people of the same age information, and this to me sounds are the most effective educators for new knowledge.’ (Participant 3, male, 16 years in focus group 2)

Different views were given by teenagers regarding the implementation of peer education about teenage pregnancy prevention programmes. Teenagers reflected that they promised by all means to teach their peers because experience is the best teacher. Research by Effendi et al. (2021) in Indonesia highlights that knowledge transfer is most effective when delivered by peers, as teenagers feel more comfortable discussing sexuality-related issues with those of the same age.

#### Sub-theme 3.2: Development of youth group

Participants felt it was essential to develop a youth group that can be continuously updated with current information to keep participants abreast of new developments within a group. Youth groups are regarded as the leaders of the future; hence, it could be an advantage to create leaders for the future through the creation of youth groups. The following were the quotes from the participants:

‘I support the vision about the development of a youth group in the sense that it would empower youth to take control of their reproductive health with one conversation at a time.’ (Participant 8, male, 19 years in focus group 1)

‘If as people of the same age can group ourselves together, together we can break down all barriers in our lives and build together a better and brighter future.’ (Participant 3, male, 15 years in focus group 1)

The establishment of youth groups plays a crucial role in empowering young individuals by providing them with opportunities for personal growth, skill development and leadership training. These groups serve as platforms for continuous learning, fostering a sense of responsibility and community amongst members. By equipping young people with the necessary knowledge and skills, youth groups help shape future leaders who can contribute positively to society. This was supported by Kashwani and Rizvi (2024) that critical skills in groups can be identified through continuing education amongst the group with the goal of creating new leaders for the future. Gleeson, Craswell and Jones (2022) believed that the sharing of experiences amongst cultural groups assisted in the formation of strong groups amongst different cultural groups of childbearing mothers. This was backed up by Gleeson et al. (2022) by supporting the statement that various cultural groups could make a positive impact towards the formation of youth groups when they are composed of members from various cultures, thus enabling them to share more of their experiences.

### Theme 4: The destiny phase: Shaping others

The destiny phase is the final stage of a youth group’s development in a teenage pregnancy prevention programme. Young people have fully embraced their potential and are now ready to shape their own self in reaching their destinies. Youth groups within teenage pregnancy prevention programmes play a vital role in shaping and empowering young individuals to become advocates for responsible decision-making and healthy relationships. By fostering peer-to-peer learning, mentorship and leadership development, these groups create a supportive environment where members can guide and influence one another positively. Through continuous education and skill-building, youth are equipped not only to make informed choices themselves but also to inspire and shape their peers, ultimately contributing to a generation that is more knowledgeable, responsible and prepared for the future. The sub-themes that emerged from the participants are as follows:

#### Sub-theme 4.1: Leadership skills

These are the skills that could be demonstrated by the leader within the group through taking an initiative such as volunteering for the proposed tasks and projects, thus demonstrating the readiness to take charge. During the teenage pregnancy prevention programme, teenagers indicated that it was beneficial to them to demonstrate leadership skills during the focus group discussions in which they would lead the group conversations within the safe and respectful environment. According to the destiny phase, participant 7, a female aged 17 years in group 4, said the following quote:

‘I never thought I could lead, but now I could see that the workshop showed me that leadership is not just about authority but is all about empowering others.’ (Participant 7, female, 17 years in focus group 4)

‘I think as a youth in this community we can fight ourselves against teenage pregnancy, thus reducing teenage pregnancy at all costs. If we could believe on ourselves as youth, looking into our abilities, that could make all our dreams come true.’ (Participant 4, female, 16 years in focus group 4)

Participants reflected that they, as young people in the community, have the power to prevent teenage pregnancy and work towards reducing it as much as possible. By believing in themselves and recognising their potential, they can achieve their dreams. This statement is consistent with the findings by Shore and Chung (2022), who reflected that leaders can either sustain or discourage work group inclusion if there is no respect amongst its members. This means that a proper leadership style is made available in an environment with utmost respect.

#### Sub-theme 4.2: Community engagement

Teenagers felt that they would appreciate teenage pregnancy prevention programmes only if the community stakeholders and parents are involved. This would help to prevent the increase in the number of teenage pregnancies; hence, through community engagement, there would be the collaboration of local organisations, such as schools, community centres, clinics and religious organisations, that could help in reaching wider audiences:

‘I think if we could engage with the nearby organizations within the communities we can as youth unlock the collective impact amongst us as youths.’ (Participant 2, female, 15 years in focus group 3)

‘I prefer members to engage themselves to the community projects and workshops as community engagement is like a thread that weaves a community together.’ (Participant 1, male, 17 years in focus group 3)

Participants indicated that they would be able to get assisted with the involvement of stakeholders in the community. The involvement of community members in teenage pregnancy prevention programmes is essential for creating a supportive and informed environment that reinforces positive change. Engaging parents, educators, healthcare providers and local leaders ensures that young individuals receive consistent guidance, mentorship and resources beyond the programme itself. A collective effort from the community strengthens the programme’s impact, promoting a culture of awareness, responsibility and support for teenagers as they navigate crucial life decisions. The findings concurred with the study conducted in Kenya by Jackson-Gibson et al. (2021), which attested that the community-based interventions of parents and stakeholders benefitted participants on teenage pregnancy prevention by working together to sustain the programme, thus helping teenagers to attend workshops and programmes that would give them a better future. This revealed that parents and community stakeholders would provide participants with accurate information regarding teenage pregnancy prevention.

#### Sub-theme 4.3: Advocacy skills

Advocacy refers to the process of promoting and supporting a particular project within the community or an organisation, especially when the project has members who could not be heard (Sager 2022). In a community, it can be done by working with communities to identify and address their needs and concerns. The participants reflected that they disseminate relevant information to the community stakeholders that is easily accessed by members within the community. The participants also discovered that it was the best time to advocate for themselves and others within the community on issues related to reproductive health.

The following are the quotes from participants:

‘With advocacy … I think if I would be advocating for the youths in a project, I would be able to be the voice of all youths in a project, hence I would be able to give relevant information as it taught me to be bold and fearless on every difficult situation.’ (Participants 2, male, 17 years in focus group 2)

‘Eish! … At first, I was too shy to talk for people in my group, but after gaining such a valuable information, it would be good for me to drive the vehicle of all youths within the community, pushing for justice for all teenagers who were challenged by gender of the opposite sex and forcing them to have sexual intercourse.’ (Participant 4, female, 16 years in focus group 4)

Almost all the participants said that community involvement would assist in the project by involving community members to be the spokespersons of teenagers so that justice could prevail amongst teenagers of the opposite sex. Community stakeholders would make the voices of the teenagers be heard, especially when they are unable to bring facts together for positive outcomes.

## Discussion of findings

Appreciative inquiry focuses on strengths-based approaches rather than problem-centred discussions. Studies such as Whitney and Cooperrider (2011) suggest that AI encourages positive engagement and motivation amongst learners by focusing on their potential rather than deficits. Applying AI in Ubuntu-infused workshops helps learners explore solutions collaboratively, fostering a proactive approach to pregnancy prevention. In consistent with the results of the previous study that assessed the impact of teenage pregnancy prevention programmes with the infusion of Ubuntu workshops, literature indicates that empowerment of self and others in Uganda played a major role in promoting the mental health of adolescent unmarried mothers, thus preventing them from suicide and major depression (Webb et al. 2023). The findings concurred with the findings in Nigeria conducted by Olorunsaiye et al. (2022), who reflected that lack of empowerment to young adults resulted in a decline in their mental status, thus failing to empower and support the mental health of others with the same challenge. This means that to support teenagers, there is a need for community structures with relevant norms within the programmes that can support adolescents with healthy adolescent sexual practices and behaviour (Viner et al. 2012). Over and above, the teenage pregnancy prevention programme has positioned teenagers to be role models in shaping their peers through access to the training programmes. This shows that there is a need for empowerment for young adults to prevent future problems of teenage pregnancy. From what was described, it was understood that teenagers got information about empowering themselves and others, which can bring positive results to teenagers. This might mean that teenagers should be made aware of the type of information from the facilitators of the programme so that they could assist them during the running of workshops.

### Limitations

The study used scenarios during focus group discussions as a method to collect data from participants because it was difficult to get participants during school periods, hence it was done on weekend days though school learners were to start with lessons at school in the morning hours and later join the training programme session in the afternoon. The study used scenarios during workshop presentations utilising focus group discussions. This method is seen as a limitation because it does not give the opportunity for the researchers to get more field notes and observational notes that are helpful for informative data collection.

## Recommendations

The findings are discussed according to the participants and the different themes as displayed. By using the AI approach, the programme can develop a comprehensive and effective teenage pregnancy prevention programme that addresses the unique needs and challenges of high school teenagers to promote a brighter future for all. The recommendations were made to nursing education, nursing practice and nursing research, as discussed under the following headings below.

### Nursing education

The authors recommend that a teenage pregnancy prevention programme should be adopted as a learning opportunity for high school learners as an alternative and formal educational pedagogy within the school curriculum. This means that high schools should benchmark on community setups to include the programmes for teenage pregnancy prevention.

### Nursing practice

It is recommended that the clinical practice environments can provide training to teenagers on sexuality health using training workshops to disseminate sexuality health messages. The providers can also use local languages to train teenagers on teenage pregnancy prevention using indigenous languages related to the reproductive health issues.

### Nursing research

The researchers recommend that the implementation of teenage pregnancy prevention should be documented so that more research can be conducted to explore further on how the programmes can be implemented to expand the body of knowledge.

## Conclusion

In conclusion, the teenage pregnancy prevention programme assisted teenagers in empowering self and others for better outcomes for the future as well as imparting knowledge that assisted in shaping others within the community. Empowering oneself is a transformative journey that unlocks immense potential for personal growth. By embracing the mindset of empowerment, the teenage pregnancy prevention programme created an environment where empowered individuals came together to drive a positive change towards teenage pregnancy prevention that helped in shaping a brighter future for all. As we empower teenagers, they become catalysts that bring a positive change in others.
